# Fourth-generation sequencing in the cell and the clinic

**DOI:** 10.1186/gm548

**Published:** 2014-04-28

**Authors:** Marco Mignardi, Mats Nilsson

**Affiliations:** 1 Department of Biochemistry and Biophysics, Science for Life Laboratory, Stockholm University, Solna Se-171 21, Stockholm, Sweden

## Abstract

Nearly 40 years ago, DNA was sequenced for the first time. Since then, DNA sequencing has undergone continuous development, passing through three generations of sequencing technology. We are now entering the beginning of a new phase of genomic analysis in which massively parallel sequencing is performed directly in the cell. Two methods have recently been described for *in situ* RNA sequencing, one targeted and one untargeted, that rely on ligation chemistry. This fourth generation of sequencing technology opens up prospects for transcriptomic analysis, biomarker validation, diagnosis and patient stratification for cancer treatment.

## The first three generations

Nucleic acid sequencing has undergone rapid and impressive development. The effect of ceaseless technological improvement is illustrated by the reduced speed and cost of recent sequencing approaches and the quantity of data being produced. It is also reflected in the way medicine is changing in the so-called ‘genomic era’, in which sequencing each person’s genome is becoming a real possibility for routine clinical practice. A very recent development is *in situ* sequencing to read nucleic acid sequences within tissues and cells [1,2]. With optimization, this approach is likely to be extremely useful in the clinic for diagnosis and personalized medicine. Such fourth-generation technology builds upon the earlier ground-breaking methods for DNA sequencing.

The first methods used to sequence DNA were described in the 1970s. The most prominent were chemical sequencing by Maxam and Gilbert [3] and the chain termination method by Sanger and colleagues [4]. Both relied on isotopic labeling of oligonucleotides and gel electrophoresis of four pools of reactions to reconstruct the DNA sequence. The first turning point in DNA sequencing came when Leroy Hood and his laboratory, in the mid-1980s, replaced radioactive labeling with fluorescent dyes and automated the Sanger method [5]. This improvement formed the basis for the development and commercialization of capillary electrophoresis sequencing machines, which allowed the assembly of the first draft complete human genome ever sequenced, in 2001.

The next turning point in DNA analysis was the introduction of second-generation sequencing machines, the so called next-generation sequencing (NGS) technologies, in 2005 [6]. Researchers moved from chain termination methods and electrophoresis separation to sequencing platforms in which the DNA starting material is generally fragmented, clonally amplified and loaded on sophisticated microchips or wells for sequencing. The Sanger method was replaced by newly developed chemistries that allow massively parallel sequencing of the human genome or transcriptome in hours to days. A third generation of sequencing methods of single nucleic acid molecules has also been introduced and promises longer, easier to map sequencing reads and lower costs. However, these true single-molecule sequencing approaches have not yet become robust enough for broad use.

## Fourth-generation sequencing

**Figure 1:**
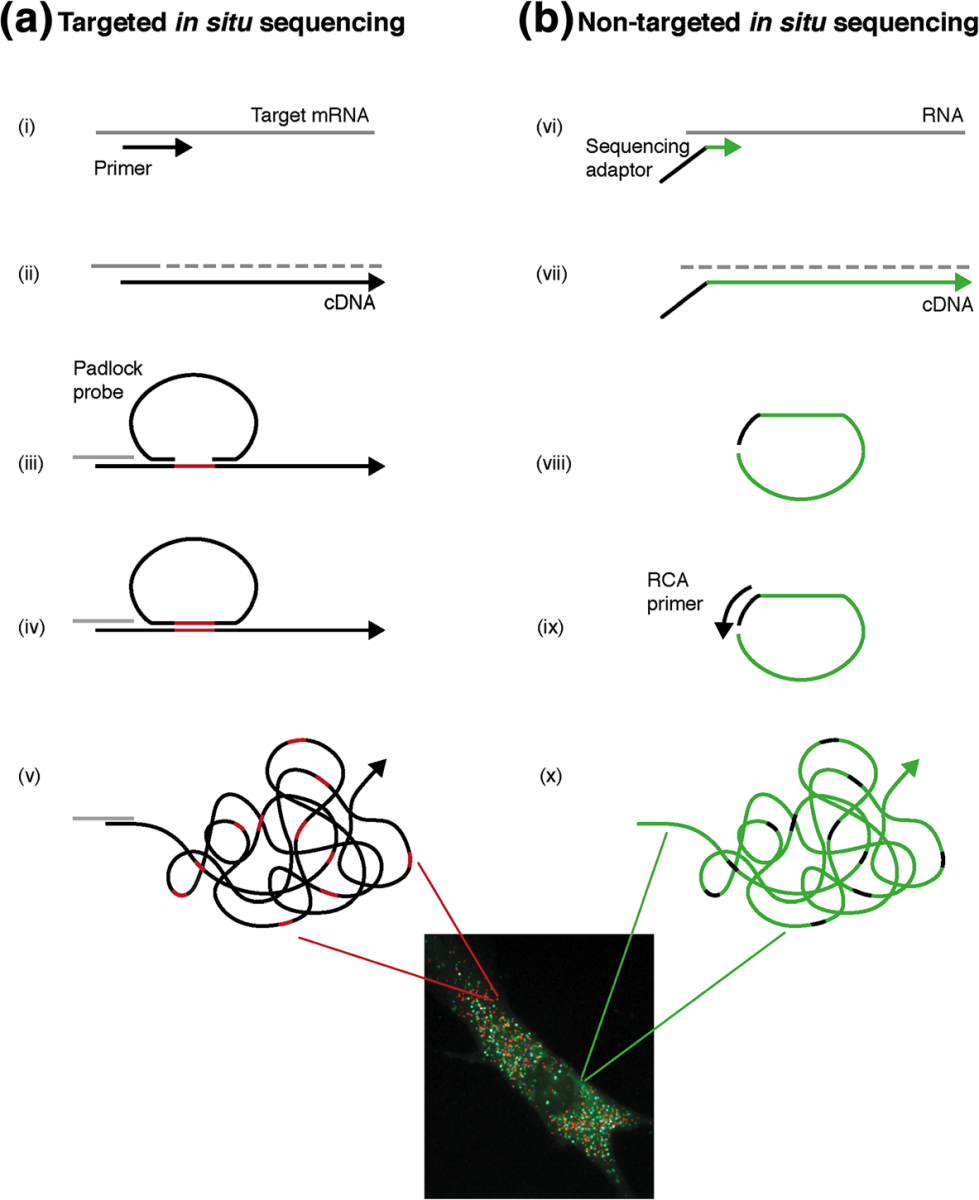
**Molecular steps to generate sequencing substrate in targeted and non-targeted *in situ *sequencing. (a)** In the targeted approach, a specific or random primer is used to retrotranscribe mRNA (gray) (i), the synthesized cDNA is made single stranded and a padlock probe is hybridized to complementary target sequences on the cDNA molecule (ii, iii), leaving a short gap between the probe arms (in red). Polymerization gap filling and DNA ligation are used to circularize the padlock probe (iv), which can be then replicated via target-primed rolling circle amplification (v). **(b)** In non-targeted *in situ* sequencing, the cDNA is synthesized by random primers containing a sequencing adaptor (vi). RNA is then digested and single-stranded cDNA molecules are self-circularized (ii, iii). An external primer is then hybridized and used to prime the rolling circle amplification (RCA) reaction (ix, x). In both the approaches, the sequencing substrate consists of a rolling circle product containing a clonally amplified region of DNA (colored in red or green), which is sequenced *in situ* by sequencing using ligation chemistries.

The recently described new (fourth) generation *in situ* sequencing method exploits second-generation NGS chemistry to read nucleic acid composition directly in fixed cells and tissues. Last year, *in situ* sequencing of mRNA was demonstrated for the first time. We used a targeted method to sequence short nucleotide sequences in breast cancer tissue sections [2]. We first generated cDNA *in situ* and then we used padlock probes, which are approximately 70-base-long oligonucleotides, to encircle a short target sequence of four to six bases (Figure 1). The gap left between the end arms of the padlock probe was filled by polymerization and sealed by a DNA ligase before being clonally amplified thousands of times via rolling circle amplification (RCA). We then used sequencing by ligation chemistry developed by Drmanac and colleagues [7] to read the target region thus cloned on the rolling circle product.

Recent work by Lee, Daugharthy and colleagues represents a step further in the development of fourth-generation technology [2]. The authors describe a novel strategy to generate amplicons in a non-targeted fashion in which random hexamers tagged with a sequencing adaptor are used to reverse transcribe RNA molecules *in situ*. The newly synthesized cDNA self-circularizes and is amplified via RCA (Figure 1). The amplicons are covalently linked to cellular proteins and can be generated in many different cell types, tissue sections and whole-mount embryos. Target mRNA molecules can be unambiguously identified thanks to the impressive read length of 27 nucleotides obtained by implementing sequencing by oligonucleotide ligation and detection (SOLiD) chemistry in the protocol [8].

The RNA-rich cellular environment poses a difficult challenge to achieving a readable density of sequencing library *in situ*. In fact, a physical limitation exists to the number of spots distinguishable at the optical resolution used by the *in situ* sequencing methods described so far. In our approach, the theoretical limitation is about 100 reads per cell and we propose strategies to go beyond this limit [2]. Church’s group overcome this limitation by partition sequencing, in which randomly mismatched sequencing primers are used to decrease read density, imaging only a pool of the total reads every cycle [1]. They achieved an average density of 400 reads per cell, half of which map to mRNA genes. Using this method, the authors show simultaneous expression of thousands of genes uniquely mapping to mRNA but also to rRNA, non-coding RNA and anti-sense RNA in fibroblasts and induced pluripotent stem cells. They further demonstrate the feasibility of their method to identify fibroblast subpopulations in a wound-healing model by detecting thousands of differentially expressed genes.

## In situ sequencing for research and diagnostics

*In situ* sequencing differs from previous sequencing generations in two respects, both of which are relevant for its use in research and diagnostics. First, the spatial distribution of the sequencing reads over the sample can be seen, adding an important level of information. We demonstrated in our article how this information can be used to visualize tissue heterogeneity based on a number of known molecular markers.

Lee and colleagues showed that subcellular resolution can be achieved with their method, an interesting feature that would allow the study of regulatory elements such as non-coding RNA species in complex populations of cells, for example, in brain tissue. A second difference is the throughput in terms of the number of cells it is possible to simultaneously analyze. Single-cell RNA sequencing methods have been developed to work robustly with just a few picograms of starting material, and there has been an increase in the rate of cell sequencing as well as a decrease in cost [9]. However, tissue material routinely used in diagnostics is composed of hundreds of thousands of cells. To dissociate and sequence single cells is technically and computationally challenging and the huge amount of information returned may not be cost effective. By sequencing RNA molecules *in situ* instead, information can be obtained by thousands of cells at the same time while maintaining single-cell or even subcellular resolution.

Although in its infancy, we foresee the use of *in situ* sequencing as a complementary tool to filter clinically relevant information from the massive amounts of data produced by traditional NGS methods, and to translate it into molecular diagnostic applications. It is likely that biomarker discovery and validation will still be done by performing deep sequencing on extracted nucleic acids. A targeted *in situ* sequencing approach may be useful instead to screen for validated biomarkers directly on routine samples while a non-targeted approach may be used to molecularly profile samples for patient stratification or molecular classification of disease. For example, at least two features make *in situ* sequencing interesting for cancer diagnostic applications. First, this method achieves single-nucleotide resolution, enabling both detection of clinically relevant point mutations, allelic variants and splice variants, and tissue expression profiling. Second, adding molecular information to classic morphological microscopic analysis allows digital and quantifiable representation of the heterogeneity of the sample, which may be important, for instance, for tumor grading and classification. It will also be useful for studying local effects, such as the contribution of the tumor microenvironment to cancer growth or tumor clonal evolution.

The two described approaches for *in situ* RNA sequencing promise exciting future prospects for research and molecular diagnostics, but further development is needed. Time, cost and the amount of manual procedure needed to perform a sequencing run are far from suitable for any clinical laboratory. A lesson learnt from NGS technologies is that, if the techniques described here hold the potential that we foresee, all these limiting factors will diminish in a few years. The implementation of *in situ* sequencing in one of the existing NGS platforms would speed up the development of these methods, enabling them to become a fundamental tool for personalized medicine.

## Abbreviations

NGS: Next-generation sequencing; RCA: Rolling circle amplification; SOLiD: Sequencing by oligonucleotide ligation and detection.

## Competing interests

The authors declare that they have no competing interests.
